# Pharmacovigilance analysis and real-world adverse event profile (safety signals and product-related issues) of potassium citrate: a US FDA adverse event reporting system (FAERS) based analysis

**DOI:** 10.1007/s00240-026-01989-0

**Published:** 2026-04-28

**Authors:** Rifat Burak Ergül, Ismail Taha Gürlek, Yasin Yitgin, Seyda Gül Ozcan, M. Fırat Özervarlı, Serdar Turan, Samed Verep, Samet Şenel, Arda Atar, Muratcan Kiremit, Amelia Pietropaolo, M. İlker Gökçe, Bhaskar Somani, Kemal Sarica, Tzevat Tefik

**Affiliations:** 1 Department of Urology, Istanbul Faculty of Medicine, Istanbul University, Istanbul, Turkey; 2 EAU Young Academic Urologists (YAU) Urolithiasis and Endourology Working Group Arnhem, Arnhem, NL-6803 The Netherlands; 3 Mus State Hospital, Mus, Turkey; 4 Department of Sports Management, Faculty of Sport Sciences, Muş Alparslan University, Mus, Turkey; 5 Istanbul Faculty of Medicine, Istanbul University, Istanbul, Turkey; 6 Demre State Hospital, Antalya, Turkey; 7 Istanbul Nisantasi University, Faculty of Medicine, Istanbul, Turkey; 8 Department of Nephrology, Cerrahpasa Faculty of Medicine, Istanbul University-Cerrahpasa, Istanbul, Turkey; 9 University of Health Sciences Taksim Training and Research Hospital, Istanbul, Turkey; 10 Department of Urology, Private Yuzyil Gebze Hospital, Gebze, Turkey; 11 Department of Urology, Ankara City Hospital, Ankara, Turkey; 12 Department of Urology, Acibadem Atasehir Hospital, Istanbul, Turkey; 13 Department of Urology, Koç University, Istanbul, Turkey; 14 Department of Urology, University Hospital Southampton NHS Foundation Trust, Southampton, UK; 15 Department of Urology, Ankara University School of Medicine, Ankara, Turkey; 16 Department of Urology, Sehit Prof. Dr. Ilhan Varank Sancaktepe Training and Research Hospital, Istanbul, Turkey; 17 Department of Urology, Biruni University Medical School, Istanbul, Turkey

**Keywords:** Potassium Citrate, Adverse Drug Reaction Reporting Systems, Pharmacovigilance, Urolithiasis, Drug Stability

## Abstract

Potassium citrate is widely used as oral alkali therapy for the prevention of recurrent nephrolithiasis. Despite its long-standing clinical use, systematically collected real-world safety data remain limited. We performed a pharmacovigilance analysis to characterize adverse event reports associated with potassium citrate using the U.S. Food and Drug Administration Adverse Event Reporting System (FAERS). FAERS reports submitted between 2014 and 2025 listing potassium citrate as the primary suspect drug were analyzed. Duplicate records were removed, and adverse events were coded using the Medical Dictionary for Regulatory Activities (MedDRA) at both the system organ class and standardized event term levels (preferred terms, PTs). Disproportionality analyses were conducted using reporting odds ratio, proportional reporting ratio, bayesian confidence propagation neural network, and multi-item gamma poisson shrinker methods. A total of 408 unique reports were included. Most reports involved adults and were physician-reported from the United States. Disproportionality analysis identified potential safety issues (signals), defined as drug event associations reported more frequently than expected in FAERS and therefore warranting further evaluation, although not establishing causality. The strongest associations involved product-related issues, laboratory investigations, and gastrointestinal disorders. When these potential safety issues were described using standardized MedDRA event terms most reflected formulation- and administration-related problems, including tablet residue in stool, abnormal solubility, swallowing difficulty, throat irritation, and foreign body sensations. Reports of rash were infrequent. This first FAERS-based analysis of potassium citrate highlights that most reported adverse events are related to formulation and administration characteristics rather than drug toxicity. These findings support the overall tolerability of potassium citrate while underscoring the importance of adherence, patient education, and formulation-specific considerations in clinical practice. As a spontaneous reporting system, FAERS is valuable for hypothesis generation, label expansion, and post-marketing surveillance, but it is not suitable for estimating the incidence or absolute risk of adverse events.

**Supplementary Information:**

The online version contains supplementary material available at 10.1007/s00240-026-01989-0.

## Introduction

Nephrolithiasis is a prevalent and increasingly common condition worldwide, affecting between 1% and 20% of the population depending on geographical and lifestyle factors [1]. Incidence has risen markedly in developed countries over the past two decades [2]. In first-time stone formers, approximately 26% recur within five years, and about half of recurrent formers have only one lifetime recurrence [3, 4]. Highly recurrent disease occurs in a little over 10% of patients [4]. Recurrent stones cause repeated renal colic/hematuria and are linked to higher risk of chronic kidney disease (CKD) and other long-term complications [5, 6].

Recent advances in surgical and endourological techniques have improved nephrolithiasis management, yet recurrence prevention remains essential and requires both lifestyle measures and pharmacological therapy [7, 8]. Among these, potassium citrate is a key pharmacological agent with well-established efficacy in reducing the recurrence risk of several major stone types [9]. As an oral alkali agent, potassium citrate is recommended as first-line therapy for the prevention of calcium stone recurrence, as well as for the management of uric acid and cystine stones by promoting urinary alkalinization and increasing urinary citrate excretion [9–11], and is also used in conditions such as distal renal tubular acidosis, chronic diarrheal states, and inherited metabolic disorders with low urinary citrate or systemic acidosis [8, 12]. It is generally preferred over sodium-based alkalis, as it lowers urinary calcium without adding sodium load [11, 13]. International guidelines designate potassium citrate as a first-line therapy for these stone types in adults and children, emphasizing individualized metabolic evaluation, urine pH monitoring, and regular follow-up to optimize outcomes [14, 15]. Despite its widespread use, most safety data come from controlled trials or limited post-marketing reports; systematically collected real-world data remain scarce, and the full spectrum of adverse events in diverse populations is yet to be fully defined.

The FDA Adverse Event Reporting System (FAERS) is a large pharmacovigilance database that compiles spontaneous adverse event reports from healthcare professionals, manufacturers, and patients. The FAERS database enables the detection of potential safety signals. In FAERS, a signal refers to a potential safety issue, a drug-event association reported more frequently than expected and therefore warranting further evaluation, although not establishing causality [16]. Reported adverse events in FAERS are coded using the Medical Dictionary for Regulatory Activities (MedDRA). Within MedDRA, a Preferred Term (PT) is a standardized term used to describe a specific adverse event, symptom, diagnosis, or clinical concept; it represents a harmonized coding term rather than the reporter’s original wording. However, as a spontaneous reporting system, FAERS is subject to underreporting and reporting bias, which may lead to incomplete representation of real-world adverse events. Accordingly, the present study aims to provide the first comprehensive characterization of potassium citrate-associated adverse events using FAERS data, thereby contributing to a more robust understanding of its safety profile and informing risk mitigation in real-world settings.

## Materials and methods

### Data source

Adverse event reports were obtained from the U.S. Food and Drug Administration Adverse Event Reporting System (FAERS) covering the period from the first quarter of 2014 (2014 Q1) to the first quarter of 2025 (2025 Q1). FAERS is structured into seven core data tables, including patient demographic and administrative information (DEMO), drug-related data (DRUG), reported adverse events coded as reaction terms (REAC), clinical outcomes (OUTC), report sources (RPSR), therapy start and end dates (THER), and reported indications for use (INDI). Additional details regarding the database structure are publicly available on the FDA website [16].

Each FAERS case is assigned a unique CASEID, which may encompass multiple follow-up submissions over time. Individual report records within a case are distinguished by a PRIMARYID, while FDA_DT denotes the date on which a specific report version was received by the FDA. Data extraction, management, and analysis were conducted using Python (version 3.13.5; Jupyter Notebook environment) with the pandas, numpy, and matplotlib libraries; Microsoft Excel was used exclusively for table preparation and formatting.

A total of 16,964,192 FAERS case reports were retrieved. Case-level deduplication was performed in accordance with FDA-recommended procedures: when multiple records shared the same CASEID, the report with the most recent FDA_DT was retained, and if both CASEID and FDA_DT were identical, the record with the highest PRIMARYID was selected [17, 18]. This process resulted in the exclusion of 2,363,307 duplicate records, yielding 14,600,885 unique cases for further screening.

**Fig. 1:**
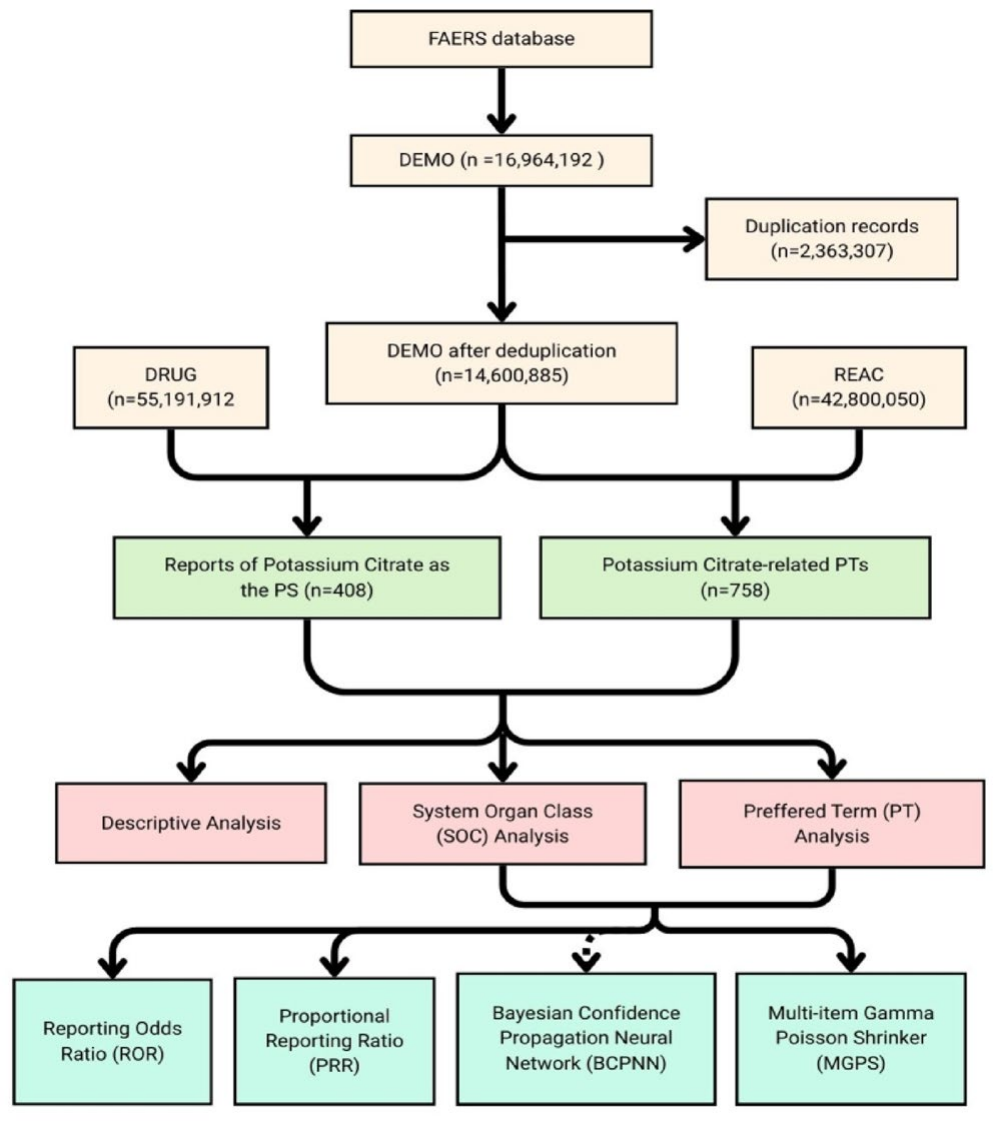
The process of extracting and analyzing Potassium Citrate-associated adverse event reports from the FAERS database. DEMO, patient demographic and administrative information; DRUG, drug information; REAC, adverse event coding; FAERS, FDA Adverse Event Reporting System

To reduce potential confounding from concomitant medications, the dataset was restricted to reports in which potassium citrate was listed as the primary suspect (PS) drug, as defined in the DRUG file [19]. This selection resulted in 408 unique FAERS reports, comprising a total of 758 adverse event entries. All reported adverse events were coded using the Medical Dictionary for Regulatory Activities (MedDRA), version 28.0, at both the Preferred Term (PT) and System Organ Class (SOC) levels. The overall data extraction and selection process is illustrated in Fig. 1.

### Statistical analysis

Disproportionality analysis is a widely used analytical approach in pharmacovigilance to identify potential associations between drugs and adverse events within large spontaneous reporting systems such as FAERS [20, 21]. This approach evaluates whether specific drug–event combinations are reported more frequently than would be expected by chance, thereby generating signals that merit further clinical or epidemiological assessment.

In this study, four complementary signal detection algorithms were applied in parallel: the Reporting Odds Ratio (ROR), Proportional Reporting Ratio (PRR), Bayesian Confidence Propagation Neural Network (BCPNN), and the Multi-item Gamma Poisson Shrinker (MGPS). ROR and PRR represent frequentist methods; ROR offers high sensitivity but may yield spurious signals in smaller datasets [22, 23], whereas PRR is considered more resilient to incomplete reporting and is routinely used by regulatory bodies such as the Healthcare Products Regulatory Agency (MHRA) and the World Health Organization (WHO) [24, 25]. In contrast, BCPNN and MGPS are Bayesian approaches designed to improve signal stability in the presence of sparse data. BCPNN incorporates prior information to enhance robustness when report counts are low [26, 27], while MGPS is particularly suited for the detection of rare events and is less influenced by data sparsity [28]. For MGPS analyses, the empirical Bayesian geometric mean (EBGM) was calculated, and uncertainty was quantified using an approximate 95% confidence interval [24]. Consistent with international recommendations, both frequentist and Bayesian methods were applied jointly to balance sensitivity and specificity in signal detection [24, 29].

Analyses were performed at both PT and SOC levels, including only events with > 3 reports (*N* > 3) [30]. Detection thresholds followed established standards [25, 26, 31, 32] (Table 1), and any PT or SOC meeting the threshold in at least two independent disproportionality methods was considered a signal. To reduce confounding by indication, PTs linked to potassium citrate’s approved uses were identified from the INDI file and excluded [28].

**Table 1:** The specific formulas and signal detection thresholds for the four disproportionality algorithms used in this study. Abbreviations: a, Number of reports containing both the target drug and the target adverse event; b, Number of reports containing other adverse events for the target drug; c, Number of reports containing the target adverse event for other drugs; d, Number of reports containing other drugs and other adverse events. 95% CI, 95% confidence interval; N, number of reports; χ², chi-squared; IC, information component; IC025, lower limit of the 95% CI of IC; E(IC), expectation of IC; V(IC), variance of IC; EBGM, empirical Bayesian geometric mean; EBGM05, lower 95% CI of EBGM; ROR, reporting odds ratio; PRR, proportional reporting ratio; BCPNN, Bayesian confidence propagation neural network; MGPS, multi-item gamma Poisson shrinker

Method	Formula	Threshold
ROR	ROR = (a × d) / (b × c) 95% CI =$\:{e}^{\left[\mathrm{l}\mathrm{n}\right(\mathrm{R}\mathrm{O}\mathrm{R})\:\pm\:\:1.96\:\times\:\:\surd\:(1/\mathrm{a}\:+\:1/\mathrm{b}\:+\:1/\mathrm{c}\:+\:1/\mathrm{d}\left)\right]}$	Lower limit of 95% CI > 1
PRR	PRR = [a / (a + b)] / [c / (c + d)] χ² = [(a × d – b × c)² × (a + b + c + d)] / [(a + b)(c + d)(a + c)(b + d)]	PRR ≥ 2, χ² ≥ 4
BCPNN	IC = log₂ [a × (a + b + c + d) / ((a + c) × (a + b))] 95% CI = E(IC) ± 2 × √V(IC)	IC₀₂₅ > 0 (lower bound of 95% CI)
MGPS	EBGM = a × (a + b + c + d) / ((a + c) × (a + b)) Approx. 95% CI =$\:{e}^{\left[\mathrm{l}\mathrm{n}\right(\mathrm{E}\mathrm{B}\mathrm{G}\mathrm{M})\:\pm\:\:1.96\:\times\:\:\surd\:(1/\mathrm{a}\:+\:1/\mathrm{b}\:+\:1/\mathrm{c}\:+\:1/\mathrm{d}\left)\right]\:}$	EBGM05 (lower 95% CI of EBGM) > 2

## Results

### Descriptive analysis

A total of 408 unique AE reports associated with potassium citrate were identified in FAERS. Demographic and clinical characteristics are summarized in Table 2. Most cases involved adults (41.7% aged 18–64 years, *n* = 170; 28.9% >64 years, *n* = 118), while 3.9% were under 18 years (*n* = 16); age was missing in 25.5% (*n* = 104). Gender distribution was 46.1% male (*n* = 188) and 42.2% female (*n* = 172). Physicians submitted 73.0% (*n* = 298) of reports, and most originated from the United States (89.2%, *n* = 364). Outcome information was available for 101 of the 408 reports (24.8%). Among all reports, the most frequent serious outcomes were other serious events (41/408, 10.0%), hospitalization (36/408, 8.8%), and death (15/408, 3.7%). A case-level summary of reports with serious outcomes is provided in Supplementary Table S1. Review of the case narratives revealed that most reports lacked sufficient clinical detail to support a direct causal relationship with potassium citrate. A large proportion of cases (79%) involved concomitant medications, and many reports were associated with multiple comorbidities or unclear indications. Additionally, several reported events, including suicide, cardiac events, and infections, are not recognized adverse effects of potassium citrate and are more likely attributable to underlying conditions or external factors.

**Table 2:** Demographic characteristics of patients with potassium citrate-associated adverse event reports in FAERS (2014Q1–2025Q1)

	*n*	%
Number of events	408	100
Age		
<18	16	3.92
18–64	170	41.67
>64	118	28.92
Unknown	104	25.49
Gender		
Male	188	46.08
Female	172	42.16
Unknown	48	11.76
Weight (kg)		
<80	10	2.45
80–100	17	4.17
>100	15	3.68
Unknown	366	89.71
Reporter		
Physician	298	73.04
Other Health Prof.	84	20.59
Consumer	26	6.37
Reported Countries (top five)		
United States	364	89.22
Unknown	22	5.39
Canada	5	1.23
Japan	3	0.74
India	2	0.49

### Signal of system organ class (SOC)

Disproportionality analysis at the SOC level identified 9 SOCs meeting the predefined criteria for a signal. Key numerical estimates are summarized below; full statistics are provided in Table 3.

**Table 3:** Statistically significant SOC-level signals for potassium citrate

SOC Name	Case Numbers	ROR(95%CI)	PRR	χ²	IC (IC025)		EBGM(EBGM05)
Product Issues	41	10.00 (7.23–13.83)	9.03	296.32		3.17 (2.85)	9.03 (6.54)
Investigations	96	6.07 (4.82–7.65)	4.79	303.95		2.26 (2.03)	4.79 (3.83)
Renal And Urinary Disorders	31	5.01 (3.47–7.24)	4.69	91.43		2.23 (1.85)	4.68 (3.25)
Gastrointestinal Disorders	60	3.34 (2.54–4.40)	2.97	82.97		1.57 (1.29)	2.97 (2.26)
Surgical And Medical Procedures	9	3.21 (1.66–6.22)	3.16	13.39		1.66 (0.99)	3.16 (1.63)
Injury, Poisoning And Procedural Complications	83	2.00 (1.57–2.55)	1.78	32.32		0.83 (0.59)	1.78 (1.40)
Metabolism And Nutrition Disorders	12	1.89 (1.07–3.37)	1.87	4.91		0.90 (0.31)	1.87 (1.05)
General Disorders And Administration Site Conditions	146	1.86 (1.51–2.28)	1.53	35.68		0.61 (0.42)	1.53 (1.26)
Cardiac Disorders	12	1.79 (1.01–3.17)	1.76	4.02		0.82 (0.23)	1.76 (0.99)

The strongest signal was observed for Product Issues (*n* = 41; ROR: 10.00, 95% CI: 7.23–13.83). Significant signals were also detected for Investigations (*n* = 96; ROR: 6.07, 95% CI: 4.82–7.65), Renal and Urinary Disorders (*n* = 31; ROR: 5.01, 95% CI: 3.47–7.24), and Gastrointestinal Disorders (*n* = 60; ROR: 3.34, 95% CI: 2.54–4.40). Other SOCs with significant disproportionality included Surgical and Medical Procedures (*n* = 9; ROR: 3.21, 95% CI: 1.66–6.22), Injury, Poisoning and Procedural Complications (*n* = 83; ROR: 2.00, 95% CI: 1.57–2.55), Metabolism and Nutrition Disorders (*n* = 12; ROR: 1.89, 95% CI: 1.07–3.37), General Disorders and Administration Site Conditions (*n* = 146; ROR: 1.86, 95% CI: 1.51–2.28), and Cardiac Disorders (*n* = 12; ROR: 1.79, 95% CI: 1.01–3.17).

A summary of the disproportionality analysis for these SOCs, including PRR, chi-squared, IC (IC025), and EBGM (EBGM05) values, is provided in Table 3.

### Signal detection at the preferred term (PT) level

Disproportionality analysis at the PT level identified 26 preferred terms (PTs) meeting the predefined criteria for a robust potential safety issue. These PTs are summarized in Table 4 and can be broadly categorized according to product characteristics and administration, perceived lack of efficacy, appropriateness of use, events related to treatment indication, and a limited number of clinical adverse effects.

**Table 4:** Statistically significant preferred terms (PTs) for potassium citrate, identified by disproportionality analysis in the FAERS database

Preferred Terms (PTs)	Case Numbers	ROR(95%CI)	PRR	χ²	IC(IC025)	EBGM(EBGM05)
Product residue present	67	695.34(533.26–906.68)	573.23	37720.8	9.14(8.88)	564.81(434.94)
Medication residue present	16	177.69(107.59–293.46)	170.27	2681.17	7.41(6.89)	169.52(102.69)
Product solubility abnormal	13	128.36(73.76–223.38)	124.01	1581.65	6.95(6.38)	123.62(71.06)
Foreign body in respiratory tract	4	109.93(40.99–294.85)	108.79	426.03	6.76(5.75)	108.49(40.45)
Foreign body	4	66.28(24.72–177.66)	65.59	254.03	6.03(5.03)	65.48(24.43)
Malabsorption	4	30.68(11.45–82.19)	30.36	113.54	4.92(3.92)	30.34(11.33)
Flank pain	4	25.87(9.66–69.31)	25.6	94.56	4.68(3.67)	25.59(9.55)
Product use complaint	8	24.89(12.36–50.16)	24.39	179.51	4.61(3.89)	24.38(12.10)
Proteinuria	7	21.37(10.11–45.14)	20.99	133.32	4.39(3.63)	20.98(9.93)
Poor quality product administered	4	18.77(7.01–50.27)	18.58	66.54	4.22(3.21)	18.57(6.93)
Product physical issue	8	18.42(9.14–37.11)	18.051	128.94	4.17(3.46)	18.04(8.96)
Product substitution issue	13	12.51(7.20–21.76)	12.12	132.98	3.60(3.03)	12.12(6.97)
Retching	4	11.02(4.11–29.51)	10.91	36.034	3.45(2.44)	10.91(4.07)
Product dispensing error	5	10.47(4.33–25.30)	10.34	42.24	3.37(2.47)	10.34(4.28)
Pollakiuria	4	5.58(2.08–14.96)	5.53	14.89	2.47(1.46)	5.54(2.07)
Throat irritation	4	5.12(1.91–13.72)	5.07	13.12	2.34(1.34)	5.08(1.90)
Drug ineffective	99	4.83(3.84–6.07)	3.83	222.5	1.94(1.71)	3.83(3.07)
Drug ineffective for unapproved indication	5	4.16(1.72–10.06)	4.12	11.86	2.04(1.14)	4.12(1.71)
Blood creatinine increased	4	3.87(1.44–10.35)	3.83	8.40	1.94(0.93)	3.84(1.43)
Abdominal discomfort	12	3.75(2.11–6.67)	3.66	23.45	1.87(1.29)	3.66(2.06)
Dysphagia	6	3.74(1.67–8.38)	3.69	11.86	1.89(1.06)	3.70(1.65)
Oropharyngeal pain	6	3.49(1.56–7.81)	3.44	10.46	1.78(0.96)	3.45(1.54)
Maternal exposure during pregnancy	5	2.76(1.14–6.68)	2.74	5.55	1.45(0.55)	2.74(1.13)
Drug dose omission	8	2.35(1.16–4.72)	2.31	6.04	1.21(0.50)	2.32(1.15)
Abdominal pain	8	2.05(1.02–4.12)	2.02	4.19	1.02(0.30)	2.02(1.01)
Rash	15	1.94(1.16–3.25)	1.89	6.52	0.93(0.40)	1.90(1.13)

The majority of high-disproportionality PTs were related to the physical characteristics and administration of the product. These included product residue present (*n* = 67; ROR: 695.34), medication residue present (*n* = 16; ROR: 177.69), product solubility abnormal (*n* = 13; ROR: 128.36), product physical issue, poor quality product administered, product substitution issue, and product use complaint. In addition, events such as foreign body and foreign body in respiratory tract were also observed among the most disproportionate PTs.

A second group of signals reflected perceived lack of efficacy of the product, including drug ineffective (*n* = 99; ROR: 4.83) and drug ineffective for unapproved indication.

Several PTs were related to potentially inappropriate or non-standard use, such as drug dose omission, product dispensing error, and maternal exposure during pregnancy.

Other reported PTs appeared to be related to the underlying indication for potassium citrate therapy, including flank pain, pollakiuria, proteinuria, and blood creatinine increased.

Only a limited number of PTs represented clinical adverse effects potentially attributable to the product itself. These included gastrointestinal or upper aerodigestive symptoms such as abdominal discomfort, abdominal pain, dysphagia, oropharyngeal pain, throat irritation, retching, and malabsorption. Rash was reported in a small number of cases (*n* = 15).

A complete summary of all significant PTs, including ROR, PRR, χ², IC (IC025), and EBGM (EBGM05) values, is provided in Table 4.

## Discussion

In this study, we conducted a pharmacovigilance analysis of potassium citrate using the FAERS database to characterize real-world adverse event reporting patterns. Identified signals were largely consistent with the known tolerability profile of potassium citrate and were predominantly related to product formulation and administration. In particular, product quality-related terms were frequently reported, reflecting issues associated with the physical characteristics and handling of the medication in routine clinical practice.

Several of the observed signals reflected complaints related to product characteristics or administration, including product residue, medication residue, abnormal product solubility, and product use complaints. Other reported terms, such as flank pain, are likely influenced by confounding by indication related to underlying stone disease. Rash and malabsorption were reported in a small number of cases and occurred infrequently. In a randomized trial of potassium–magnesium citrate, treatment discontinuation due to rash was reported in a single patient, supporting biological plausibility while underscoring the rarity of this finding [9]. Cardiovascular-related signals appeared weak and inconsistent, despite their inclusion in product labeling. As with all spontaneous reporting systems, these findings should be interpreted with caution and regarded as hypothesis-generating rather than confirmatory.

A considerable proportion of reported signals were related to product characteristics and administration, including product residue present, medication residue present, abnormal product solubility, and product use complaints. Most reports originated from the United States, where potassium citrate is commonly available as extended-release oral tablets. According to FDA-approved labeling, these formulations may occasionally cause mild gastrointestinal discomfort and can pass through the gastrointestinal tract with intact wax-matrix remnants visible in the feces, representing an expected formulation characteristic rather than a dissolution failure [33]. Accordingly, some residue-related FAERS reports likely reflect anticipated product behavior rather than true defects. The high daily tablet burden required for urinary alkalinization may nevertheless contribute to swallowing difficulty or patient-perceived tolerability issues. Consistent with this, swallowing difficulty was reported in approximately 11% of participants in a randomized trial of potassium–magnesium citrate, with one treatment discontinuation, and gastrointestinal complaints were common [9]. In another randomized trial of potassium citrate (*n* = 57), mild gastrointestinal symptoms occurred in 17% of participants, and 31.6% discontinued therapy, predominantly because of non-compliance rather than intolerance, possibly related to pill burden (mean ≈ 9 tablets/day) [10].

Formulation-related factors such as tablet size, excipients, and dissolution rate may influence gastrointestinal transit and patient tolerability, potentially contributing to perceptions of reduced effectiveness. A Cochrane systematic review on citrate salts for calcium-containing kidney stones highlighted substantial heterogeneity in salt types, dosages, and formulations across trials, complicating identification of the most effective and best-tolerated preparation [11]. In a long-term follow-up of sodium–potassium citrate therapy, adherence rates were 68% among patients with recurrence and 57% among those without recurrence, with only 38% remaining on treatment at evaluation [34]. Similarly, Basiri et al. reported that patients intolerant to potassium citrate powder experienced adverse effects such as salty taste, epigastric pain, nausea, and heartburn; after switching to tablets, patient satisfaction improved markedly, with Visual Analog Taste Scale scores decreasing from 8.35 to 2.0 (*P* = 0.0001) [35]. Taken together, these observations indicate that formulation and administration factors may play an important role in tolerability and adherence during long-term citrate therapy.

From a clinical perspective, these findings underscore the value of individualized management to support adherence and tolerability. Clinicians may consider screening for swallowing difficulties, reinforcing correct administration and hydration in accordance with labeling, and arranging early follow-up when patients report residue or perceived lack of effectiveness [13, 34, 35]. Ongoing post-marketing surveillance and incorporation of patient-reported outcomes may further help to clarify formulation-related tolerability issues during long-term therapy.

Oral alkali agents are among the most frequently prescribed therapies for the prevention of recurrent nephrolithiasis, and potassium citrate remains a cornerstone of long-term medical management because of its broad indications and established efficacy. In the context of our FAERS analysis, the observed reporting patterns primarily highlight formulation- and administration-related tolerability considerations rather than unexpected safety concerns. Awareness of these practical issues, together with patient education on correct administration and hydration, may help support adherence during long-term therapy and optimize clinical outcomes.

The identification of serious outcomes reported in FAERS, including hospitalization or death, should be interpreted with caution. A review of case-level data for these 84 unique serious reports (Supplementary Table S1) did not reveal a consistent clinical pattern suggestive of potassium citrate-related toxicity. Reported events were heterogeneous and encompassed a wide range of clinical scenarios. Although a small number of reports included hyperkalemia-related preferred terms, these were infrequent and did not occur in a consistent or dominant pattern across serious cases. Given the descriptive nature of FAERS data and the absence of complete clinical narratives and temporal relationships, these findings do not support a clear or biologically plausible association between potassium citrate and serious adverse outcomes. As such, these reports should be interpreted as hypothesis-generating rather than evidence of causality.

This study has several limitations inherent to spontaneous reporting systems such as FAERS. First, adverse event submissions are voluntary and originate from heterogeneous sources; therefore, under-reporting, delayed reporting, and misreporting or incomplete information may bias disproportionality estimates. Second, FAERS lacks exposure denominators and a defined user population, precluding calculation of incidence or absolute risk. Third, even when reports are complete, causality cannot be established: disproportionality signals indicate statistical association rather than proof of cause–effect and may be influenced by confounding by indication, co-medications, and stimulated reporting following regulatory safety communications or labeling changes. Consequently, serious outcomes reported in FAERS, including hospitalization or death, should be interpreted with caution. Fourth, records can include duplicates or fragmented submissions despite de-duplication, and coding variability can affect event classification. Fifth, key covariates (e.g., dose, treatment duration, renal function, comorbidities) are often missing or inconsistent, limiting confounding control. Sixth, the predominance of U.S. reports may constrain generalizability. Finally, the modest number of potassium-citrate cases reduces precision for rarer events and the stability of some estimates over time. Overall, these findings should be interpreted as hypothesis-generating and warrant confirmation in complementary study designs with defined exposure denominators, formulation-specific data, and improved confounding control. Future studies integrating longitudinal datasets and patient-reported outcomes may further clarify the influence of formulation and administration factors on tolerability and adherence in routine clinical practice.

## Conclusion

This pharmacovigilance analysis of potassium citrate in FAERS is the first to characterize its AE profile using this database. Overall, the reported events were largely consistent with the established tolerability profile of citrate therapy and were predominantly related to formulation and administration factors that may affect patient experience and adherence. Rash was reported infrequently and warrants further evaluation in complementary data sources. Further studies with defined exposure denominators and formulation-specific information are needed to better contextualize these observations.

## Supplementary Information

Below is the link to the electronic supplementary material.

Supplementary Material 1

## Data Availability

The data is publicly available and can be found here: [https://www.fda.gov/drugs/surveillance/fdas-adverse-event-reporting-system-faers] (https:/www.fda.gov/drugs/surveillance/fdas-adverse-event-reporting-system-faers) . Detailed Python code is available upon reasonable request from the authors.

## Declarations

### Generative AI and AI-assisted Technologies

During the preparation of this work, the authors used AI in order to improve the readability and language of the manuscript. After using this tool, the authors reviewed and edited the content as needed and takes full responsibility for the content of the publication.
