# Supplementary material for: Pharmacovigilance analysis and real-world adverse event profile (safety signals and product-related issues) of potassium citrate: a US FDA adverse event reporting system (FAERS) based analysis
Source: Urolithiasis. 2026 Apr 28;54(1):95. doi: 10.1007/s00240-026-01989-0 (PMC13124964; doi:10.1007/s00240-026-01989-0)
Supplement: Supplementary file 1 — Supplementary Material 1 [file 240_2026_1989_MOESM1_ESM.docx]

| Case ID | Outcome(s) | Reported PT(s) | Reported Case Detail |
| --- | --- | --- | --- |
| 17500414 | Death | Completed suicide | Product used for unknown indication; concomitant medications reported |
| 17501328 | Death | Adverse drug reaction | Product used for unknown indication; concomitant medications reported |
| 18154964 | Death | Adverse drug reaction | Product used for unknown indication; concomitant medications reported |
| 21325235 | Death | Localised infection | Indication reported as cystinuria; concomitant medications reported |
| 22768579 | Death | Death | Product used for unknown indication; no additional clinical detail available |
| 22961540 | Death | Death | Indication reported as cystinuria; concomitant medications reported |
| 23627892 | Death | Death | Concomitant tiopronin reported; no additional clinical detail available |
| 21791521 | Death, Hospitalization | Drug ineffective | Multiple concomitant medications reported; multiple comorbid conditions reported |
| 22524439 | Death, Hospitalization | Drug ineffective | Indication reported as hypokalaemia; no additional clinical detail available |
| 22915504 | Death, Hospitalization | Drug ineffective | Indication reported as hypokalaemia and electrolyte imbalance; concomitant medications reported |
| 23712837 | Death, Hospitalization | Drug ineffective | Indication reported as ventricular fibrillation and ventricular tachycardia; concomitant medications reported |
| 24135489 | Death, Hospitalization | Drug ineffective | Indication reported as hypokalaemia; concomitant medications reported |
| 24782193 | Death, Hospitalization | Drug ineffective | Indication reported as supplementation therapy; concomitant medications reported |
| 24782197 | Death, Hospitalization, Other Serious | Anti-neutrophil cytoplasmic antibody positive vasculitis | Indication reported as Graves' disease; concomitant medications reported |
|  |  |  |  |
| 13859173 | Disability | Abdominal pain, diarrhoea, rash, urticaria, nephrolithiasis | Product used for unknown indication; concomitant medications reported |
| 20774026 | Disability | Skin weeping, urticaria, blister, pruritus | Indication reported as renal lithiasis prophylaxis; concomitant medications reported |
| 21830698 | Disability | Arthritis, arthralgia, knee deformity, arthropathy | Indication reported as nephrolithiasis; no concomitant medications reported |
| 22181217 | Disability, Hospitalization, Life-Threatening, Required Intervention | Chest pain, anxiety, drug hypersensitivity, sepsis, multiple laboratory abnormalities | Indication reported as hypocitraturia and nephrocalcinosis; concomitant medications reported |
|  |  |  |  |
| 14624322 | Hospitalization | Respiratory symptoms, product quality issues | Indication reported as nephrolithiasis; concomitant medications reported |
| 14651409 | Hospitalization | Product solubility abnormal | Indication reported as nephrolithiasis; concomitant medications reported |
| 15863199 | Hospitalization | Asthma, cardiac failure, fluid retention | Indication reported as cystinuria; concomitant medications reported |
| 17205385 | Hospitalization | Blood potassium decreased | Product used for unknown indication; no concomitant medications reported |
| 17349626 | Hospitalization | Abdominal discomfort, confusional state | Indication reported as nephrolithiasis; concomitant medications reported |
| 17742531 | Hospitalization | Urinary tract infection | Indication reported as nephrolithiasis; no concomitant medications reported |
| 18132458 | Hospitalization | Neurotoxicity, mental status changes | Indication reported as renal tubular acidosis; concomitant medications reported |
| 18233141 | Hospitalization | Movement disorder, muscle symptoms | Blood potassium decreased reported; no concomitant medications reported |
| 19194249 | Hospitalization | Cardiac events, suicide attempt | Product used for unknown indication; no concomitant medications reported |
| 19238426 | Hospitalization | Cardiomyopathy, incorrect administration | Suicide attempt reported; no concomitant medications reported |
| 21187336 | Hospitalization | Transient ischaemic attack | Indication reported as cystinuria; concomitant medications reported |
| 21375869 | Hospitalization | Renal pain, Drug ineffective | Indication reported as nephrolithiasis and cystinuria; concomitant medications reported |
| 21525613 | Hospitalization | Drug ineffective for unapproved indication | Indication reported as adrenocortical carcinoma; concomitant medications reported |
| 21525615 | Hospitalization | Hyperkalaemia, off-label use | Indication reported as hypokalaemia; no concomitant medications reported |
| 21525616 | Hospitalization | Drug ineffective | Indication reported as multiple conditions; concomitant medications reported |
| 21700942 | Hospitalization | Drug ineffective | Indication reported as cystinuria; concomitant medications reported |
| 21781999 | Hospitalization | Drug ineffective | Indication reported as cystinuria; concomitant medications reported |
| 21854498 | Hospitalization | Drug ineffective | Indication reported as cystinuria; concomitant medications reported |
| 21995668 | Hospitalization | Flank pain, Drug ineffective | Indication reported as cystinuria; concomitant medications reported |
| 23596068 | Hospitalization | Chest pain | Concomitant medications reported; no additional clinical detail available |
| 23726501 | Hospitalization | Drug ineffective | Indication reported as cystinuria and nephrolithiasis; concomitant medications reported |
| 23742022 | Hospitalization | Drug ineffective | Indication reported as ureterolithiasis; concomitant medications reported |
| 24575177 | Hospitalization | Drug ineffective | Indication reported as cystinuria; concomitant medications reported |
| 24674227 | Hospitalization | Pulmonary congestion, dyspnoea | Indication reported as hypokalaemia and hypernatraemia; concomitant medications reported |
| 24746204 | Hospitalization, Death | Treatment failure, noncompliance | Indication reported as Graves' disease; concomitant medications reported |
| 24019152 | Hospitalization, Life-Threatening | Hyperkalaemia, incorrect dosing | Concomitant medications reported; no additional clinical detail available |
| 21085533 | Hospitalization, Other Serious | Diabetic neuropathy, drug interaction | Product used for unknown indication; concomitant medications reported |
| 23734894 | Hospitalization, Other Serious | Hypokalaemia, renal tubular acidosis | Indication reported as epilepsy; concomitant medications reported |
|  |  |  |  |
| 13896958 | Other Serious, Life-Threatening | Cough, foreign body in respiratory tract, product physical issue | Concomitant medications reported; laboratory abnormalities reported |
| 10426254 | Other Serious | Blood glucose decreased, tremor, prostatic pain | Indication reported as nephrolithiasis prophylaxis; concomitant medications reported |
| 12316977 | Other Serious | Abdominal distension, diarrhoea, vomiting, muscle spasms | Indication reported as renal lithiasis prophylaxis; no concomitant medications reported |
| 14065233 | Other Serious | Arthritis, diarrhoea, metabolic acidosis | Indication reported as haematuria; concomitant medications reported |
| 15148861 | Other Serious | Hyperkalaemia | Concomitant medications reported |
| 15423215 | Other Serious | Erythema, pemphigus, rash, skin lesions | Indication reported as hyperuricaemia; concomitant medications reported |
| 15473543 | Other Serious | End stage renal disease, transplant dysfunction | Indication reported as hyperoxaluria; concomitant medications reported |
| 15479578 | Other Serious | End stage renal disease | Indication reported as hyperoxaluria; concomitant medications reported |
| 16469723 | Other Serious | Palpitations | Indication reported as nephrolithiasis; concomitant medications reported |
| 16500387 | Other Serious | Oral burning, stomatitis, hypogeusia | Indication reported as nephrolithiasis; concomitant medications reported |
| 16668553 | Other Serious | Drug ineffective, Product substitution issue | Indication reported as nephrolithiasis; no concomitant medications reported |
| 16697537 | Other Serious | Burning sensation, petechiae, chemical burn | Indication reported as nephrolithiasis; concomitant medications reported |
| 16861277 | Other Serious | Gastrointestinal haemorrhage | Indication reported as nephrolithiasis; no concomitant medications reported |
| 17955832 | Other Serious | Infant sedation, off-label use | Indication reported as seizure; concomitant medications reported |
| 18042651 | Other Serious | Choking, product quality issue | Product used for unknown indication; no concomitant medications reported |
| 18072305 | Other Serious | Product consistency issue, vomiting, treatment noncompliance | Indication reported as renal lithiasis prophylaxis; concomitant medications reported |
| 19893046 | Other Serious | Dysphagia, product size issue | Product used for unknown indication; no concomitant medications reported |
| 19991120 | Other Serious | Drug ineffective | Indication reported as cystinuria; no concomitant medications reported |
| 19991122 | Other Serious | Urticaria, drug ineffective, off-label use | Indication reported as renal tubular acidosis; concomitant medications reported |
| 19991124 | Other Serious | Nephrolithiasis, hypercalciuria, drug ineffective | Indication reported as hypocalcaemia; concomitant medications reported |
| 20817873 | Other Serious | Dyspepsia, dyspnoea, product issue | Indication reported as nephrolithiasis; no concomitant medications reported |
| 21283846 | Other Serious | Aortic valve replacement, drug ineffective | Indication reported as cystinuria; concomitant medications reported |
| 21330779 | Other Serious | Product contamination, product quality issue | Product used for unknown indication; no concomitant medications reported |
| 21652114 | Other Serious | Blood glucose increased | Indication reported as renal lithiasis prophylaxis; concomitant medications reported |
| 22128787 | Other Serious | Atrioventricular block, acute kidney injury, hyperkalaemia | Indication reported as hypertension; concomitant medications reported |
| 22144557 | Other Serious | Acute kidney injury, atrioventricular block, hyperkalaemia | Indication reported as hypertension; concomitant medications reported |
| 22243454 | Other Serious | Blood potassium increased/decreased, diarrhoea, neoplasm | Product used for unknown indication; concomitant medications reported |
| 22516852 | Other Serious | Arrhythmia, off-label use | Indication reported as hypokalaemia; concomitant medications reported |
| 23074300 | Other Serious | Renal function decline, metabolic disorder | Indication reported as cystinuria; concomitant medications reported |
| 23322490 | Other Serious | Drug ineffective, off-label use | Indication reported as nephrolithiasis; concomitant medications reported |
| 24429429 | Other Serious | Pregnancy exposure, abortion missed | Product used for unknown indication; concomitant medications reported |
| 24553617 | Other Serious | Drug ineffective | Indication reported as nephrolithiasis; no concomitant medications reported |
| 24553671 | Other Serious | Drug ineffective for unapproved indication | Indication reported as swelling; concomitant medications reported |
| 24678275 | Other Serious | Premature delivery, maternal exposure | Indication reported as hypokalaemia; no concomitant medications reported |
| 24678276 | Other Serious | Foetal exposure, premature birth | Product used for unknown indication; no concomitant medications reported |
| 24705032 | Other Serious | Chronic kidney disease, drug ineffective | Indication reported as primary hyperoxaluria; no concomitant medications reported |
| 24705033 | Other Serious | Chronic kidney disease, drug ineffective | Indication reported as primary hyperoxaluria; no concomitant medications reported |
| 12572028 | Other Serious, Life-Threatening | Arrhythmia, bundle branch block, hyperkalaemia | Indication reported as urinary calculus; no concomitant medications reported |

**Supplementary Table S1.** Case-level summary of FAERS reports with serious outcomes in which potassium citrate was reported as the primary suspect drug
